# No effect of glucose administration in a novel contextual fear generalization protocol in rats

**DOI:** 10.1038/tp.2016.183

**Published:** 2016-09-27

**Authors:** L Luyten, N Schroyens, K Luyck, M S Fanselow, T Beckers

**Affiliations:** 1 Research Group Psychology of Learning and Experimental Psychopathology, Faculty of Psychology and Educational Sciences, KU Leuven, Leuven, Belgium; 2 Research Group Experimental Neurosurgery and Neuroanatomy, KU Leuven, Leuven, Belgium; 3 Department of Psychology, University of California, Los Angeles, Los Angeles, CA, USA; 4 Department of Psychiatry and Biobehavioral Sciences, University of California, Los Angeles, Los Angeles, CA, USA

## Abstract

The excessive transfer of fear acquired for one particular context to similar situations has been implicated in the development and maintenance of anxiety disorders, such as post-traumatic stress disorder. Recent evidence suggests that glucose ingestion improves the retention of context conditioning. It has been speculated that glucose might exert that effect by ameliorating hippocampal functioning, and may hold promise as a therapeutic add-on in traumatized patients because improved retention of contextual fear could help to restrict its generalization. However, direct data regarding the effect of glucose on contextual generalization are lacking. Here, we introduce a new behavioral protocol to study such contextual fear generalization in rats. In adult Wistar rats, our procedure yields a gradient of generalization, with progressively less freezing when going from the original training context, over a perceptually similar generalization context, to a markedly dissimilar context. Moreover, we find a flattening of the gradient when the training-test interval is prolonged with 1 week. We next examine the effect of systemic glucose administration on contextual generalization with this novel procedure. Our data do not sustain generalization-reducing effects of glucose and question its applicability in traumatic situations. In summary, we have developed a replicable contextual generalization procedure for rats and demonstrate how it is a valuable tool to examine the neurobiological correlates and test pharmacological interventions pertaining to an important mechanism in the etiology of pathological anxiety.

## Introduction

Anxiety disorders are associated with significant disability and poor quality of life; however, their pathophysiological mechanisms are only beginning to be understood, and also in terms of treatment there is still great room for improvement.^1^ It has been proposed that in-depth studies of the neurobiology of anxiety may open up new treatment avenues.^2, 3^ Pavlovian fear-conditioning procedures (contextual or cued fear conditioning) are valuable tools in this regard.^4, 5, 6^ Brain regions involved in both types of conditioning are partly, but not entirely, the same, with the dorsal hippocampus having a role in contextual, but not cued fear conditioning. Moreover, conditioning to a complex, unpredictable context may be pertinent to several anxiety disorders, which entail rather diffuse, free-floating anxiety (for example, generalized anxiety disorder, post-traumatic stress disorder (PTSD) and panic disorder).^7, 8^ Our focus is therefore on contextual generalization, that is, generalization of contextual fear conditioning.

Generalization, or the spreading of fear from actual threat signals to instances that merely resemble them, is a core characteristic of anxiety disorders and a key element of what makes them so disabling.^9, 10, 11^ Its clinical importance is underlined by recent studies showing excessive generalization in anxiety patients.^12, 13, 14, 15^ Contextual generalization, for example, avoiding all dark alleys because you were once assaulted in such context and because similar environments now cause extreme anxiety, appears to be a hallmark of PTSD.

Generalization research is gaining momentum; however, until now, contextual generalization has been largely neglected (but see refs 16, 17, 18). A behavioral protocol to study contextual generalization in rodents should allow us to investigate its neurobiological correlates and pharmacological treatment options, with more invasive techniques than those that can be used in humans. This paper will first discuss the development of a contextual generalization protocol for rats and, second, the use of this new procedure to examine the effects of systemic glucose administration on contextual generalization.

In a series of experiments, we aimed to develop a contextual generalization protocol with a robust gradient, that is, a strong fear response in a previously shocked context A, an intermediate fear response in a perceptually similar context B and a low fear response in a dissimilar context C. Such a gradient would imply that rats are able to discriminate context B from the original training context A (A>B), but nevertheless show substantial freezing in B, resulting from perceptual contextual generalization, not from mere sensitization (B>C). We opted for a ‘pure' contextual generalization protocol, without explicit discrimination training. Such a procedure may be more ecologically valid, because in real life, often there will be no repeated, alternating confrontations with perceptually similar safe and dangerous contexts. Moreover, training with (for example, explicitly dangerous context A and safe context C) versus without discrimination learning may result in non-negligible differences in the amount, or even nature, of contextual generalization in context B.^19, 20, 21^ We also investigated the effect of a prolonged interval between acquisition and test, and hypothesized a flattening of the generalization gradient, with elevated anxiety in generalization context B after a longer interval. Likewise, many anxiety patients experience a gradual increase in the number of stimuli or situations that elicit anxiety.^9, 22^

Next, we examined the effect of systemic glucose administration on contextual generalization with this novel protocol. Recently, Glenn *et al.*^23^ proposed glucose as an easy-to-use addition to the treatment of anxiety. Prior animal research had already shown that glucose (often administered at a dose of 250 mg kg^−1^) can influence several memory and anxiety tasks,^24, 25, 26, 27, 28^ and that such tasks are associated with hippocampal glucose changes.^26, 29^ These hippocampus-dependent tasks may deplete the available glucose, and this depletion may then be reversed by glucose administration.^30^ In a 2-day human fear-conditioning study, Glenn *et al.*^23^ found that glucose (versus placebo) ingestion, following acquisition, resulted in better retention of contextual, but not cued fear. They argued that this improved retention, which they interpreted as resulting from a more specific and detailed contextual memory, might reduce ‘overgeneralization' and thereby the chance of developing long-term emotional problems, like PTSD. However, in contrast to the prediction of Glenn *et al.*, a stronger retention of the original (traumatic) context memory might as well increase, instead of decreasing, the generalization to novel but similar contexts, which would be an unwanted side effect when using glucose in a clinical population. With our generalization protocol, we can address this important, unanswered question and investigate whether memory specificity is indeed enhanced by glucose administration. To increase the comparability of both our studies, we adopted many of their procedural details, including 2 h of fasting before the experiment, immediate glucose administration after the acquisition session and a 24-h training-test interval.

Note that if systemic glucose administration effectively influences hippocampal functioning, this may indeed result in effects on contextual generalization, as prior research already implicated the hippocampus in contextual discrimination and generalization.^18, 31, 32, 33, 34, 35^ In addition, stress may affect hippocampal functioning and thereby increase generalization in anxiety patients.^36^ Furthermore, the strong evidence for hippocampal aberrations in anxiety disorders, such as PTSD,^37^ supports the possible involvement of this brain region in pathological processes such as generalization.^12^

## Materials and methods

Male Wistar rats (±300 g at the time of training, obtained from Janvier Labs, Saint-Berthevin, France) were used for all experiments, which were approved by the KU Leuven animal ethics committee, in accordance with the Belgian Royal Decree of 29 May 2013 and European Directive 2010/63/EU. Animals were housed in pairs in cages with cage dividers and maintained on a 14 h/10 h light/dark cycle. All experimental sessions were meticulously scheduled using free ExpTimer software.^38^

First, we optimized a contextual generalization procedure for rats (Experiments 1–3) and then we examined the effects of systemic glucose administration (Experiments 4–5) in this novel protocol.

### Experiment 1

**Figure 1:**
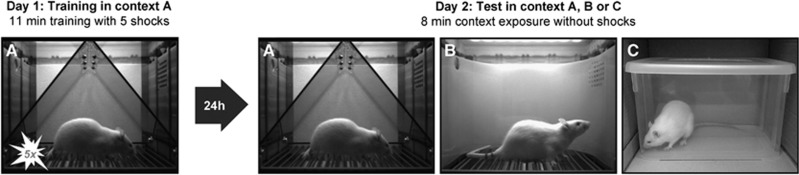
Behavioral protocol. On Day 1, male Wistar rats are trained in context A. After 4 min of acclimation, they receive five footshocks (0.8 mA—1 s, 90 s interval). One minute later, they are put back in their home cage. On Day 2, rats are tested in either the original conditioning context A, a similar generalization context B or a more dissimilar context C. During this 8-min test, no shocks are administered. Afterward, the percentage freezing during the test session is scored manually from videos.

On Day 1, rats were trained in context A (Figure 1). Four minutes after the start of the session, they received five unsignaled footshocks (0.8 mA, 1 s), separated by 90 s. One minute after the last shock, animals were returned to their home cage. Twenty-four hours later, half of the rats were tested in context A and the other half in similar context B (*n*=16 per group). During this test, rats were exposed to the context for 8 min without shocks. For an overview of all experimental designs, see Supplementary Figure S2. Freezing during training was measured with VideoFreeze software (Med Associates, Fairfax, VT, USA) and rats were block-randomized into groups with comparable post-shock freezing levels (in Experiments 1–3). Freezing during test was measured manually by a trained observer (continuous measurement with a stopwatch from video recordings), as previous findings indicated that comparison of software-scored freezing in different contexts was not reliable.^39^ Percentage freezing was calculated as the percentage of time the rat was freezing during the 8-min test on Day 2. Data are from one observer in Experiment 1, and the average of two observers in all other studies.

Context A (Figure 1) consisted of a standard chamber (Med Associates), with a standard grid floor, a black triangular ‘A-frame' insert, illuminated by infrared and white light (intensity level 5) and cleaned and scented with a household cleaning product. Context B (Figure 1) consisted of a standard chamber, with a staggered grid floor, a white plastic curved back wall insert, infrared light only and was cleaned and scented with another cleaner. Each chamber was located in one of two identical sound-attenuating boxes.

### Experiment 2

Procedures were identical to Experiment 1, except that rats were divided into three groups (*n*=8 per group), one of which was tested in dissimilar context C on Day 2 in order to obtain a generalization gradient.

Context C (Figure 1) consisted of a transparent plastic container (34 × 25 × 20 cm), placed in a cardboard box (80 lux), in the same room as contexts A and B. The plastic container was cleaned with alcohol before and after every session. A webcam (Logitech, Lausanne, Switzerland) was placed inside the cardboard box to record the freezing behavior during test.

### Experiment 3

Procedures were identical to Experiment 2, except that the interval between training and test was 8 days instead of 1 day (*n*=8 per group).

### Experiment 4

Procedures were identical to Experiment 2, except for these changes: (1) food was taken away starting 2 h before each session and returned about 35 min after the end of each session. (2) Immediately after the animal was taken out of the training box on Day 1, a drop of blood was collected from the tail vein while the animal was gently restrained by another experimenter. Blood glucose levels were assessed using a glucometer (Aviva, Roche, Rotkreuz, Switzerland). This measurement was repeated 30 min later and also immediately after the test on Day 2. (3) Immediately after the first blood glucose measurement, animals received an intraperitoneal injection of glucose (250 mg kg^−1^, 1 g glucose dissolved in 16 ml saline) or saline (*n*=10 per group, but data from one glucose animal tested in context B were lost because of technical difficulties). Group sizes were comparable to those of prior behavioral studies demonstrating effects of systemic glucose administration.^26, 27, 28^ Observers were blinded to the group allocation (glucose or vehicle) while scoring freezing behavior.

### Experiment 5

Procedures were identical to Experiment 4, except for these changes: (1) rats were not only food-deprived, but water was also taken away during periods of food deprivation. (2) Glucose (250 mg kg^−1^, 1 g glucose dissolved in 8 ml purified water) was administered orally using a 1-ml syringe while another experimenter held the animal (*n*=9 per group). Control animals received the same volume of water (*n*=5 per group). To habituate the rats to this drinking procedure, all rats had one practice session 3 days before the experiment, during which they were given 0.5 ml of the glucose solution.

### Statistical analyses

Data were analyzed using Statistica 12 (StatSoft) with unpaired *t*-tests (Experiment 1), one-way (Experiments 2 and 3), factorial (Experiments 4 and 5) or repeated-measures (Experiments 4 and 5) analyses of variance (ANOVAs) with Tukey's *post hoc* tests where appropriate. Assumptions were met. Graphs were made with GraphPad Prism v.4.03 (GraphPad Software, La Jolla, CA, USA).

## Results

In an extensive series of studies (data not shown), conducted both at KU Leuven and UCLA (University of California, Los Angeles, CA, USA), we aimed to optimize a behavioral protocol for contextual generalization in rats. Although numerous published fear conditioning studies include a ‘different context' as a control condition, there is rarely an intention to investigate contextual generalization in itself. Here, we endeavored to develop a protocol with a contextual generalization gradient (A>B>C; Figure 1). In our first experiments, we included startle as well as freezing as behavioral measures (*cf.* Luyten *et al.*^40^), but we obtained high and comparable startle responses in all contexts; therefore, we abandoned this approach^41^ (see Supplementary Information). We tried within-subject designs, with rats being tested in more than one context, but encountered non-negligible test order effects. In addition, counterbalancing of contexts A and B was not possible because of unequal acquisition when using different grid floors.^39^ The use of Long-Evans rats, which may perform better on discrimination tasks,^42, 43^ instead of Wistar rats, did not improve our results. Finally, we succeeded in fine-tuning the contextual characteristics and training parameters, thereby developing a robust contextual generalization gradient (Experiments 1 and 2).

### Experiment 1: Discrimination between conditioning context A and generalization context B

On Day 1, rats showed very low-baseline freezing in context A during 4 min of acclimation (mean±s.d.: 1±1%), followed by post-shock freezing during the last 7 min of the training session, that is, starting from the first footshock (mean±s.d.: 49±17%).

**Figure 2:**
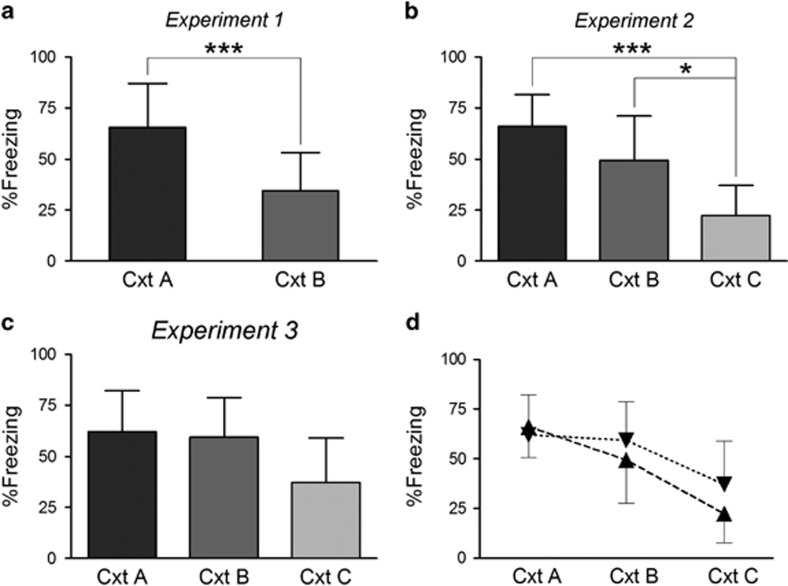
Contextual generalization gradients. %Freezing (mean and s.d.) during the 8-min test in Experiments 1, 2 and 3. (**a**) Significant discrimination between contexts A and B (*n*=16 per group), ***P<0.001, unpaired *t*-test. (**b**) Generalization gradient with a gradual decrease in freezing from A to B to C (*n*=8 per group), *P<0.05, ***P<0.001, Tukey's *post hoc* tests. (**c**) Flatter generalization gradient with longer interval between training and test (8 days instead of 1 day; *n*=8 per group). (**d**) Illustrative comparison of generalization gradients with 1- and 8-day intervals. No statistical analyses were conducted because data are taken from two separate experiments (Experiment 2, with 1-day interval: ▴ and Experiment 3, with 8-day interval: ▾). Visual inspection of the graphs indicates no incubation of fear in context A, and more generalization in contexts B and C with the 8-day interval. Cxt, context.

During the test session on Day 2, freezing in the generalization context B was significantly lower than in the original training context A (*t*(30)=4.35, *P* <0.001), indicating that rats discriminated between both contexts (Figure 2a). As intended, there remained considerable freezing in context B (mean±s.d.: 34±19%), supporting a substantial transfer of anxiety from context A to B.

### Experiment 2: Contextual generalization gradient with contexts A, B and C

To further extend our behavioral protocol, one-third of the rats were now tested in context C (Figure 2b). The main aim of adding this more dissimilar context was to show that freezing in context B was not merely the consequence of nonspecific sensitization, but, at least partially, the result of a perceptual generalization process. The data confirmed this hypothesis, as freezing in context C was lower than that in context B. The one-way ANOVA showed a main effect of Context (F(2,21)=12.75, *P*<0.001), and Tukey's *post hoc* tests revealed significantly less freezing in context C relative to A (*P*<0.001) and to B (*P*<0.05).

### Experiment 3: Flatter contextual generalization gradient with prolonged training-test interval

As an additional check of the validity of our behavioral protocol, the interval between training and test was prolonged with 1 week (Figure 2c). If the previously found gradient A>B>C was indeed a contextual generalization gradient, and not just the accidental result of specific contextual characteristics, we should expect a flattening of the generalization gradient over time because of the forgetting of perceptual contextual attributes.^22^ Note that the data from Experiments 2 and 3 (Figure 2d) should be compared with caution, as they originate from two separate studies, which were, however, conducted and analyzed by the same experimenters. In addition, post-shock freezing during training was virtually the same in both studies (42±15% in Experiment 2 and 44±19% in Experiment 3). Keeping these considerations in mind, the data do support a broader generalization gradient in Experiment 3, with no discrimination between contexts A and B (62±20% freezing in A and 59±19% in B), because of increased freezing in B, while freezing in A remained stable as compared with the 1-day interval. Finally, there also appeared to be more freezing in context C with the longer interval.

### Experiment 4: Effect of post-training glucose injection on the contextual generalization gradient

**Figure 3:**
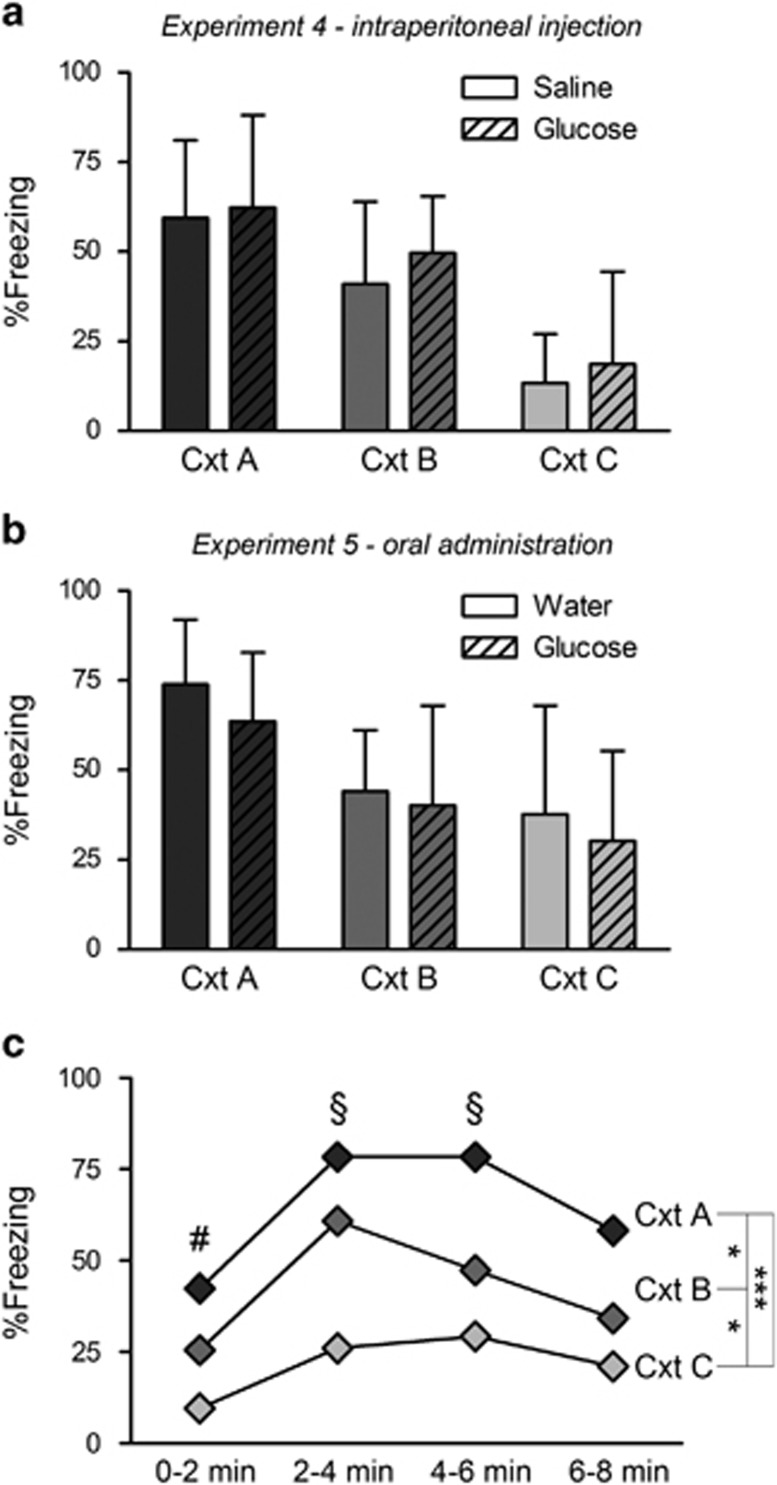
Effect of post-training glucose on contextual generalization. %Freezing (mean and s.d.) during the 8-min test in Experiments 4 and 5. (**a**) No effect of post-training intraperitoneal glucose injection (250 mg kg^−1^) on the contextual generalization gradient (*n*=9–10 per group). (**b**) No effect of post-training oral glucose administration (250 mg kg^−1^) on the contextual generalization gradient (*n*=5 per water group and *n*=9 per glucose group). (**c**) Average %freezing during test is shown in four 2-min blocks for the control rats (saline and water) of Experiments 4 and 5 combined (*n*=15 per context). There is increased freezing toward minute 4, especially in contexts A and B, presumably indicating time-locked shock expectancy (first shock was delivered after 4 min on the training day). When collapsing data from all contexts, we find ^#^less freezing during the first 2 min than during the three following two-min blocks (*P*<0.0001, *P*<0.0001, *P*<0.05, respectively) and ^§^more freezing during the two middle blocks than during the last block (*P*<0.0001 and *P*<0.01, respectively). We also replicate the previously found contextual generalization gradient: A>B>C, *P<0.05, ***P<0.001, Tukey's *post hoc* tests. Cxt, context.

Building upon the intriguing findings of Glenn *et al.*, we hypothesized that post-training glucose administration might reduce the amount of contextual generalization, without necessarily affecting the degree of contextual freezing in the original training context (Figure 3a). Blood glucose levels immediately after training were 127±20 mg dl^−1^, with no group differences. Thirty minutes after intraperitoneal glucose/saline injection, blood glucose levels were slightly increased/decreased, but not significantly. Post-training glucose injections did not affect generalization the next day. A factorial ANOVA showed no main effect of Drug and no Context × Drug interaction. We did find a main effect of Context (F(2,53)=22.34, *P* <0.0001), thereby replicating the generalization gradient observed in Experiments 1 and 2.

### Experiment 5: Effect of post-training oral glucose administration on the contextual generalization gradient

In the next study, we aimed to investigate whether oral administration of glucose versus water would have an effect on the generalization gradient (Figure 3b). Blood glucose levels immediately after training were 116±11 mg dl^−1^, with no group differences. Thirty minutes after glucose/water administration, blood glucose levels were slightly increased, especially in glucose rats, but not to a significantly different extent (see Supplementary Information for more details). Again, post-training glucose administration did not affect generalization the next day. A factorial ANOVA showed no main effect of Drug and no Context × Drug interaction. There was, however, a main effect of Context (F(2,36)=7.52, *P* <0.01), indicating the presence of a generalization gradient.

In Experiments 5 and 6, we also analyzed freezing in four subsequent 2-min blocks to inspect the time course of freezing throughout the test (Figure 3c). For this analysis, we only included the control groups from both studies (saline and water rats). A repeated-measures ANOVA with Greenhouse–Geisser correction showed main effects of Context (F(2,42)=14.78, *P* <0.0001) and Block (F(2.54,106.69)=24.46, *P* <0.0001), but no significant interaction. Tukey's *post hoc* tests showed a replication of the contextual generalization gradient (A>B>C), and increased freezing toward minute 4 and in the following minutes, presumably indicating time-locked shock expectancy, as shocks were given from minute 4 onward during training.

## Discussion

In a series of experiments, we developed a contextual fear generalization procedure for rats and examined the effect of systemic glucose interventions in this new protocol.

Given the clinical relevance of generalization for anxiety disorders,^44^ this behavioral protocol creates opportunities^45^ for in-depth investigations of this insufficiently understood phenomenon. As there is ample evidence for distinct neural correlates of contextual and cued fear conditioning,^5, 46, 47, 48^ it will be interesting to examine the differences between their respective generalization.^11^ Moreover, from a clinical point of view, it seems relevant to look into the transfer of anxiety for complex, unpredictable situations to similar contexts, rather than only focusing on generalization of fear for specific, predictable cues, as is the case with generalization of cued fear conditioning. This increased interest is also reflected by the recent development of a contextual generalization protocol for human fear conditioning,^16^ after the successful cued fear generalization procedure of Lissek *et al.*^49^

To the best of our knowledge, this is the first report of a ‘pure' (that is, without discrimination training) contextual generalization protocol for rats, with a freezing decrement during test, demonstrating a downward generalization gradient from the original training context (A) over a similar (B) to a dissimilar (C) context. An earlier study already described post-shock freezing in three increasingly different contexts, but without the crucial long-term retention test after at least 1 day of consolidation and without a gradient from A to B to C.^50^ Here, we presented the optimization and repeated replication of a robust generalization protocol for rats. In addition, we found evidence for a flattening of the contextual generalization gradient over time, which is in line with previous findings,^17, 18, 22, 51, 52^ and indicates that previously discriminable contexts become more functionally interchangeable over time. We also found that freezing during test followed the time course of the training session, especially in test contexts A and B, with increased freezing around minute 4, which might reflect some type of time-bound shock expectancy.^53^

Using this new protocol, we addressed several questions that arose from the interesting observation that post-training glucose administration resulted in better retention of contextual fear in a human fear-conditioning procedure.^23^ Here, we aimed to investigate whether glucose administration would affect generalization of contextual fear. Although glucose was given orally in the human study, we decided to first use systemic administration via intraperitoneal injection in Experiment 4, as all prior research on behavioral effects of glucose used injections for acute glucose administration,^24, 25, 26, 28^ and because it is generally assumed that the oral route is not workable for rodent research.

The absence of any effects of glucose injection in our study might be explained by the dose that was used, although a literature review indicated that 250 mg kg^−1^ should be adequate to obtain behavioral effects in rats and that higher doses (400–500 mg kg^−1^) might be ineffective.^25, 26, 28^ In addition, although interspecies comparison is difficult, our dose was in the same order of magnitude as the 25 g of glucose dose that was given in the human study (that is, 333 mg kg^−1^ for a participant of 75 kg).^23^

Note that Glenn *et al.* did not infer the superior retention of contextual memory from a direct comparison of the levels of fear in the conditioned context between the glucose versus placebo groups, but used a differential fear-conditioning procedure instead. Therefore, it is difficult to make predictions about the expected effect in our context A. One could argue that the ‘easy' task of remembering context A would not require additional glucose and is therefore already at an optimal level. Alternatively, one could expect more freezing in context A in glucose versus control rats as a sign of stronger retention. It is possible that we were unable to find such an increase because of a ceiling effect, as we observed relatively high freezing in context A in control rats, or because systemic glucose is insufficient to intervene with truly fearful memories, as discussed below. Nevertheless, we expected an effect of glucose in context B. Glenn *et al.* proposed that glucose may improve the retention of contextual fear by making it more specific, which could have therapeutic value in PTSD patients. However, in our study, we did not find any evidence for glucose having such a protective effect against generalization.

To exclude that the lack of effect on contextual generalization was specific to the route of administration, we switched to oral administration^54^ of the glucose solution versus water in Experiment 5. One of the reasons why we also tested the oral intake was to increase the comparability with the human fear-conditioning study. Glenn *et al.* tried to exclude an influence of sweetness by using a saccharin placebo, but it remains possible that there are some, perhaps time-limited, effects of oral glucose, which are bypassed by intraperitoneal injection. For example, oral glucose (but not saccharin) induces a rapid rise in plasma insulin before nutrient absorption, which is not seen with systemic injection,^55, 56^ and taste cells directly transfer information to the brain through ATP and serotonin transmission.^57^ We did not find any effects of oral glucose on contextual generalization in our rats either.

Note that there are additional procedural differences between our experiments and those conducted by Glenn *et al.* For example, we used a simpler conditioning procedure without discrimination training and we used the pure vehicle (saline or water) as a control condition. However, we feel that the most important difference is the degree of stress that was induced by the behavioral protocol. Although a human fear-conditioning procedure might generate some arousal, it is implausible that it induces ‘real' anxiety, as reflected by the relatively low subjective arousal ratings throughout such studies.^23, 58^ It is generally assumed that rats in a conditioning procedure do experience actual fear or anxiety. In other words, as already stated by the authors of the human glucose study, it is possible that exogenous glucose (at a 250–333 mg kg^−1^ dose) is not sufficiently powerful to affect contextual fear in a ‘real' traumatic context. Our extra blood glucose measurements (see Supplementary Information) seem to support this notion. Furthermore, the handling of the rats required for the glucose or vehicle administration and tail vein punctures probably resulted in the release of stress hormones,^59, 60^ with accompanying hyperglycemia, which may have overshadowed any memory-enhancing effects of exogenous glucose.

In an additional experiment (see Supplementary Information), we manipulated the dorsal hippocampus directly, as a first step to shed some light on its role in contextual generalization in our protocol. Unexpectedly, low overall freezing levels hampered the interpretation of these data; however, our findings did not support the suggestion by Glenn *et al.* that impaired hippocampal functioning would result in less specific contextual memories.

In summary, our data question the applicability of systemic glucose to reduce generalization of fearful/traumatic memories.

## Conclusion

There is still much to be explored in the field of contextual fear generalization. Such findings may enhance our insights in anxiety disorders, which are often characterized by disabling generalization. Here, we have developed a new and replicable contextual generalization protocol for rats. Using this procedure, we examined the generalization-reducing effects of glucose put forward by other authors in search of new therapeutic approaches for traumatized patients. Such effects were not endorsed by our data. To conclude, our novel behavioral protocol is a valuable translational tool to test pharmacological approaches, conduct neurobiological studies or scrutinize findings from the human literature.
