# Supplementary material for: No effect of glucose administration in a novel contextual fear generalization protocol in rats
Source: Transl Psychiatry. 2016 Sep 27;6(9):e903–. doi: 10.1038/tp.2016.183 (PMC5048216; doi:10.1038/tp.2016.183)
Supplement: Supplementary Information [file tp2016183x1.pdf]

Supplement

No effect of glucose administration in a novel contextual fear generalization protocol in rats

Laura Luyten, Natalie Schroyens, Kelly Luyck, Michael S. Fanselow, Tom Beckers

Table of Contents

Piloting a Protocol with Combined Startle and Freezing Measurements 2

Overview of the Experimental Designs 4

Experiment 5: Supplementary Glucose Results 5

Experiment 6: Materials and Methods 7

Experiment 6: Results and Discussion 8

Supplementary References 10

Piloting a Contextual Fear Generalization Protocol with Combined Startle and Freezing Measurements

In a series of three studies, we piloted a contextual fear generalization protocol, including both freezing and startle responses as behavioral indices[1]. These experiments with male Wistar rats (n = 64) were set up by analogy with previous studies[2,3] in which freezing was scored during the first 5 minutes of the test session (i.e., before startle probes were given), followed by a sequence of short loud noises (100-dB, 50-ms white noise) to elicit startle reflexes. Note that all studies were conducted using a single Med Associates Acoustic Startle Reflex chamber of which the contextual features were changed in between rats.

Study I

Context A was a small (rectangular or cylindrical, counterbalanced) cage with a grid floor, scented with vanilla odor and lit by a dim red house light in an otherwise dark box. Context B consisted of the same cage, but without the grid, with a plastic floor and without the odor. Context C was a different cage (cylindrical or rectangular), without a grid, with a plastic floor and without an artificial odor.

On Day 1, rats were pre-exposed to context A, B and C for 8 min per context. On Day 2, rats were conditioned to context A with 10 unpredictable shocks (0.8 mA – 250 ms) (30 min session). On Day 3, all rats were tested for expression of contextual fear in context A (8 min session). On Day 4, one third of the rats was tested in context A, one third in context B and one third in context C (20 min session). Startle probes were presented on Day 1 (5 probes), Day 3 (5 probes) and Day 4 (30 probes).

The weights of the rectangular and cylindrical cages differed, thereby influencing the startle measurements. Thus, the raw startle amplitudes of the different groups could not be compared directly. Therefore, the startle measures from this study are presented as %generalization, i.e., average startle on Day 4 in context X divided by average startle on Day 1 in context X. Likewise, the freezing data from this study are corrected for baseline freezing, i.e., %freezing on Day 4 minus Day 1 (Fig. S1).

Study II

Context A was a rectangular cage with a grid floor, an odor (vanilla or rose, counterbalanced) and a dim colored light (red in right corner or blue in left corner, counterbalanced) in an otherwise dark box. Context B consisted of the same cage, but without the grid, with a plastic floor and with the other odor and light.

On Day 1, rats were pre-exposed to context A and B for 11 min per context (one context before noon and one after noon). On Day 2, rats were conditioned to context A with 10 unpredictable shocks (0.8 mA – 250 ms) (30 min session). The control group received no shocks. On Day 3, all rats were tested for expression of contextual fear in context A (11 min session). On Day 4, half of the rats was tested in context A before noon and in context B after noon, and vice versa for the other half (11 min per context). Ten startle probes per context were given on Day 1, Day 3 and Day 4. Startle amplitudes and %freezing on Day 4 are displayed in Fig. S1. Note that, due to large test order effects, only data from the first test context on Day 4 are shown.

Study III

Contexts A and B were identical to those used in Study II. Context C was a large Plexiglas bowl with a layer of bedding.

On Day 1, rats were pre-exposed to context A, B and C for 21 min per context. Pre-exposure to the 3 contexts was separated by 6-10 min and the second context was always context C. On Day 2, rats were conditioned to context A with 10 unpredictable shocks (0.8 mA – 250 ms) (30 min session). On Day 3, all rats were tested for expression of contextual fear in context A (11 min session). On Day 4, half of the rats was tested in context A and context C (21 min sessions separated by 7 min, counterbalanced order). The other half was tested in context B and context C. Startle probes were given on Day 1 (20 probes in context A and B), Day 3 (8 probes in context A) and Day 4 (20 probes in context A or B). Probes could not be delivered in context C because it was positioned outside of the Startle Reflex chamber. Startle amplitudes and %freezing on Day 4 are shown in Fig. S1.

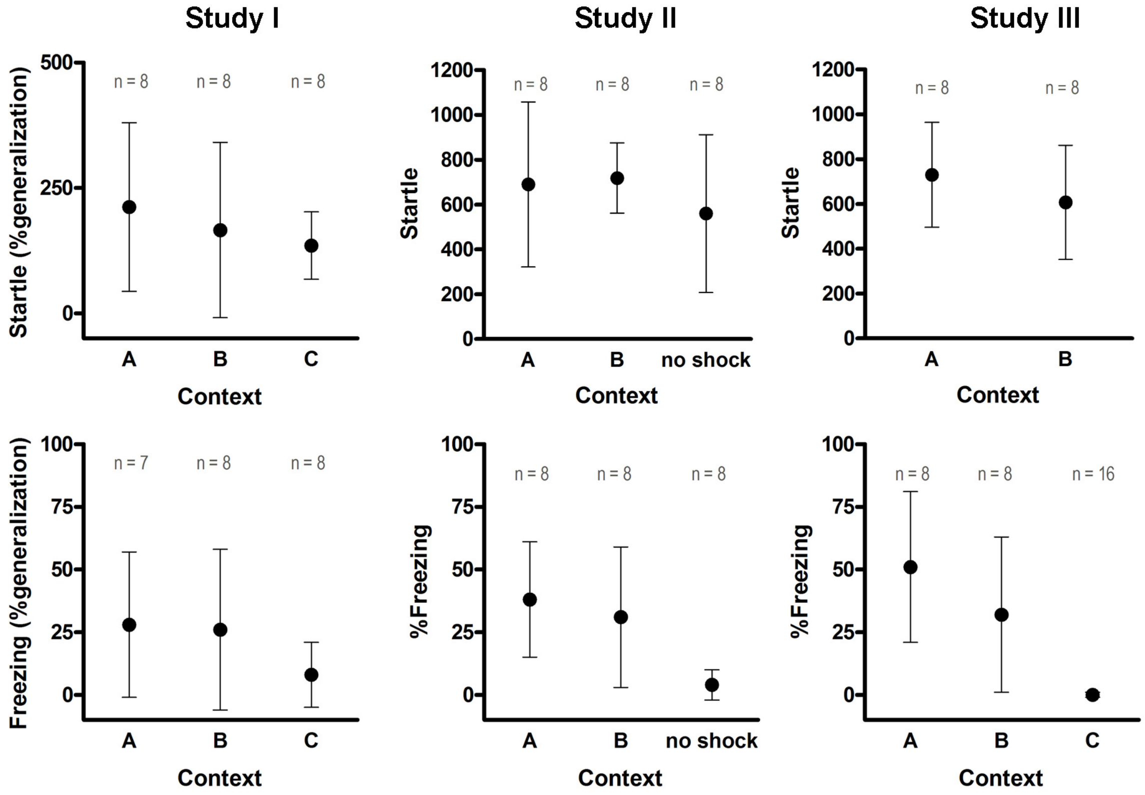

Figure S1: Three pilot studies with combined startle (upper panels) and freezing (lower panels) measurements. Means ± standard deviations are shown.

Conclusion

In these first pilot experiments, we included startle as well as freezing as behavioral measures, but we obtained high and comparable startle responses in all contexts (upper panels in Fig. S1). The considerable amount of generalization may be partially explained by the salient and relatively aversive nature of the startle probes, which were presented in all test contexts (except for context C in the third study), or the fact that animals were pre-exposed to all contexts (to allow for habituation of the startle response). Based upon the results of these three pilot studies, we abandoned the startle response approach, and only included freezing measurements during further optimization of the contextual generalization protocol, which is described in the main text.

Overview of the Experimental Designs

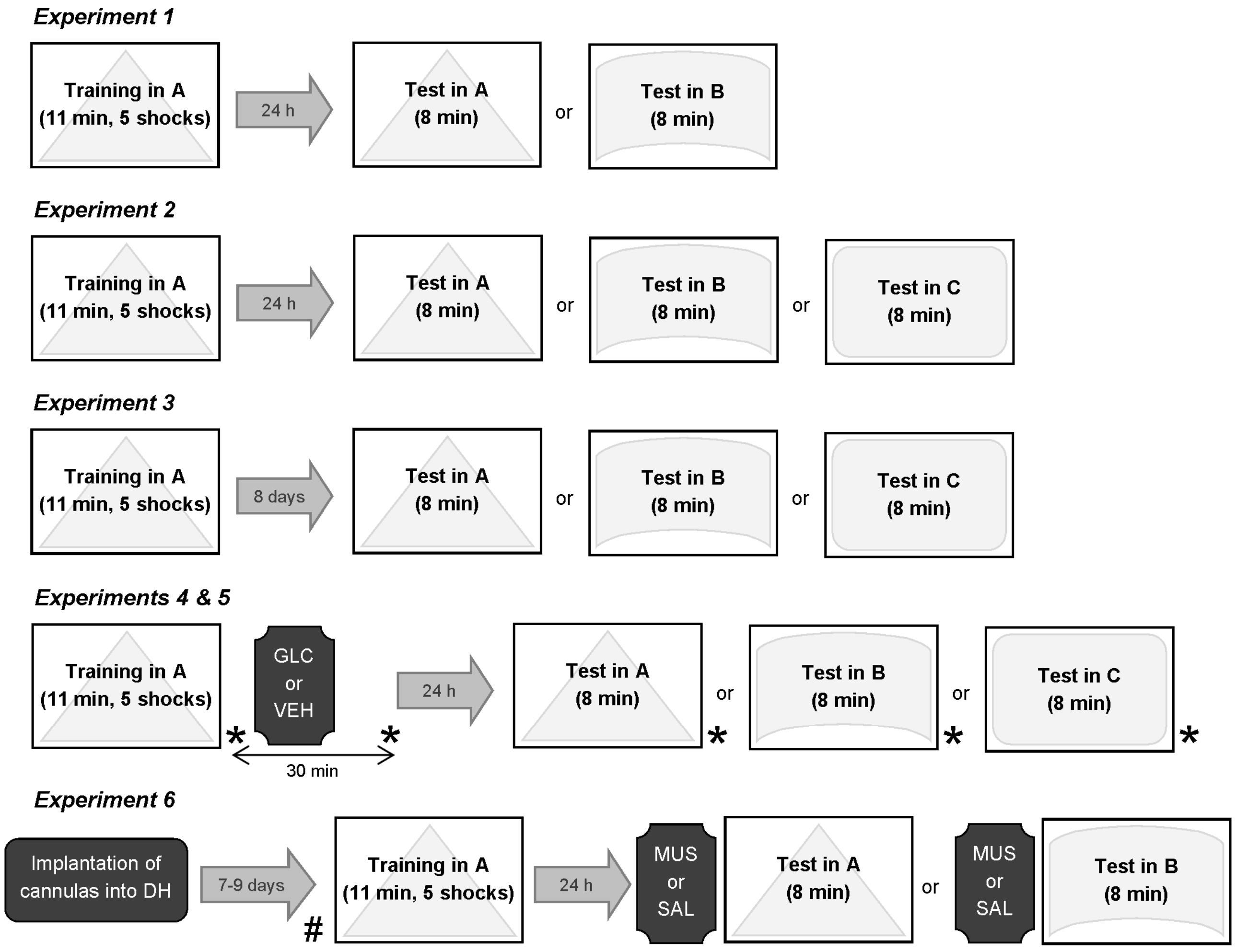

Figure S2: Experimental designs*.* *GLC = glucose (250 mg/kg) administration (intraperitoneal (IP) in Experiment 4 and oral in Experiment 5), VEH = vehicle administration (IP saline in Experiment 4 and oral water in experiment 5), * collection of blood drop from tail vein for glucose measurement, DH = dorsal hippocampus, # sham infusion ±45 min before training, MUS = muscimol (0.5 µg per hemisphere) infusion or SAL = saline infusion ±45 min before test.*

Experiment 5: Supplementary Glucose Results

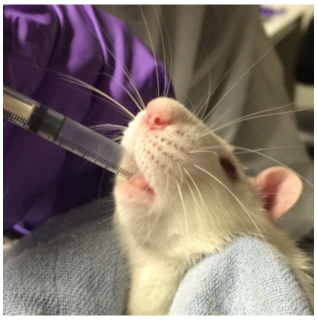

Figure S3: Oral administration with 1-ml syringe.

In Experiment 5, food and water was taken away during 2 hours prior to the training session. Immediately after the 11-min training session, blood glucose was measured in a drop of blood collected from the tail vein, immediately followed by glucose or water administration (***Fig. S3***) and a second blood glucose measurement 30 minutes after the first one.
The rather long 30-minute interval was mainly chosen not to further interfere with immediate consolidation processes after training, which may be influenced by a quickly following, somewhat stressful, second tail vein puncture.
The 30-min measurement was merely added as a manipulation check, and we expected elevated blood glucose levels in the glucose rats, but not in the water rats. However, the data from Experiment 5 (***Fig. S4A***) showed that there was also a (non-significant) increase in the water rats, albeit slighter than in the glucose group. On an individual level, about 80% of the rats in both glucose and water groups showed elevated blood glucose levels.

To further investigate this and to gain more insight into the time course of the blood glucose changes, we conducted two additional tests using 28 rats from Experiment 5. These tests were carried

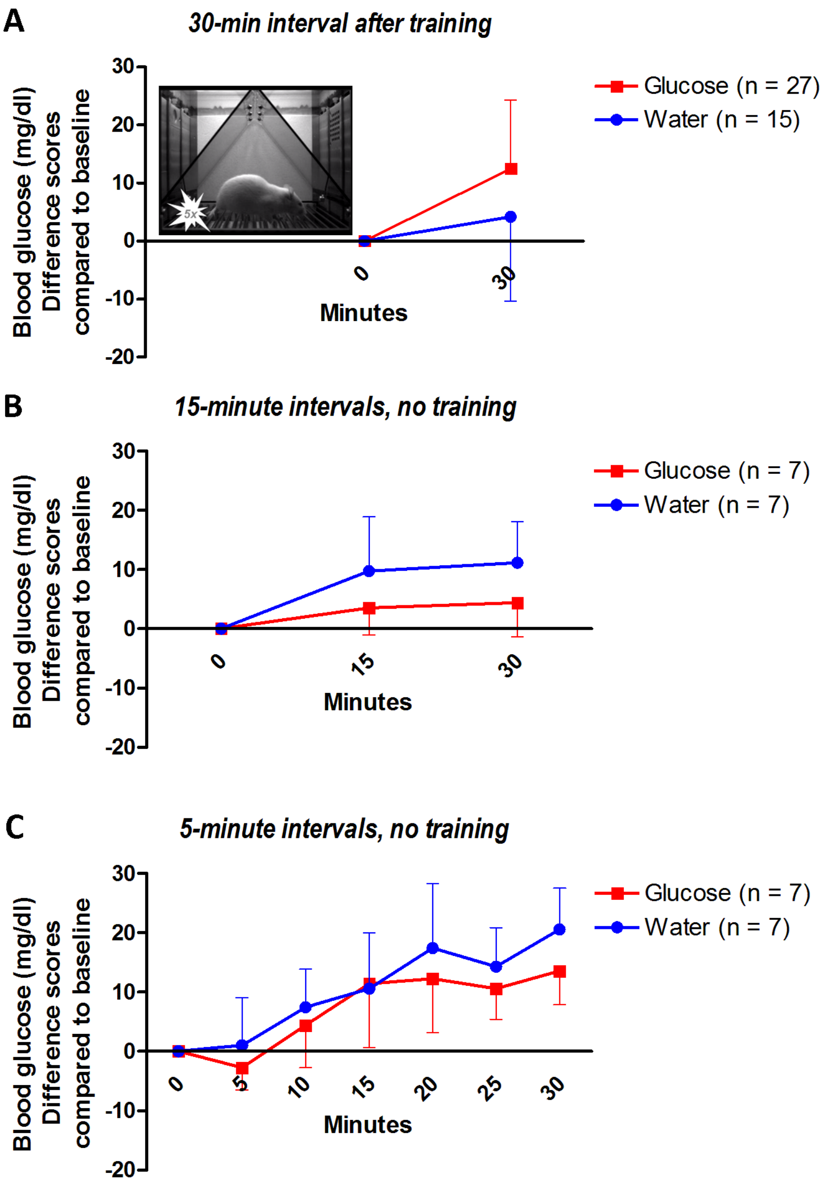

Figure S4: Blood glucose levels after oral glucose (250 mg/kg) or water administration. *Difference scores (mean and standard deviation) are shown, i.e. the increase or decrease as compared to baseline (minute 0).*

out one week later, in another room to minimize generalization from the preceding experiment. Rats were food- and water-deprived starting 131 minutes (i.e., 2 hours + the duration of the 11-min training session) before the first blood sampling, after which glucose or water was administered orally. Blood glucose was then monitored for 30 minutes in 15-minute intervals (***Fig. S4B***) or in 5-minute intervals (***Fig. S4C***).

Baseline blood glucose levels at minute 0 were comparable for all tests (***Table S1***). A repeated-measures ANOVA on the 15-minute interval data (difference scores compared to baseline) showed a significant effect of Drug ($F(1,12) = 8.96$, $p = .01$) with higher blood glucose levels in water (!) rats than in glucose rats (Tukey's posthoc test, $p = .01$) after oral administration. A similar trend was observed in the 5-minute interval data (difference scores compared to baseline), although this could not be substantiated statistically. Note that oral glucose administration induces a very rapid rise in plasma insulin (even prior to nutrient absorption). This might (partially) explain the lower blood glucose levels that we found in the glucose animals as compared with the water animals[4,5]. The repeated-measures ANOVA on the 5-minute interval data showed a significant effect of Time ($F(5,60) = 11.70$, $p < .0001$), indicating an overall increase in blood glucose levels in both groups. Taken together, these data seem to indicate that the blood sampling and handling of the animal in itself was a stressor[6,7] that may have resulted in a release of endogenous glucose[8], which seemed to overrule any effect of glucose administration, at least at a dose of 250 mg/kg. These data also suggest that an earlier second blood glucose measurement would not have given a more clear-cut manipulation check in Experiment 5.

Experiment 5 (30-min interval) *(Fig. S4A)*	
Glucose groups	114 (± 10) mg/dl
Water groups	120 (± 13) mg/dl
Extra test with 15-min intervals *(Fig. S4B)*	
Glucose group	120 (± 6) mg/dl
Water group	117 (± 9) mg/dl
Extra test with 5-min intervals *(Fig. S4C)*	
Glucose group	115 (± 7) mg/dl
Water group	114 (± 6) mg/dl

Table S1: Baseline blood glucose levels (at minute 0). *Blood glucose (mean ± standard deviation) after 131 min of food and water deprivation was comparable in all tests, irrespective of whether rats had just received 5 unpredictable footshocks or not.*

Although we were not sure whether the 30-minute interval would allow us to detect an increase in blood glucose after exogenous glucose administration, previous experience from ourselves and others[2,9] appeared to indicate that even very low doses of injected glucose result in increasing cerebral glucose levels, about 30 min after administration. However, it is possible that blood, as opposed to cerebral, glucose levels would have returned to baseline after 30 min, especially when using a relatively low dose (i.e. 250 mg/kg as opposed to e.g. 2 g/kg which is used in glucose tolerance tests[10]). Moreover, it has been previously observed that there might be differences between cerebral glucose levels and those measured in peripheral blood[11,12].

Finally, for exploratory purposes, we also measured blood glucose levels immediately after the test session (as indicated in ***Fig. S2***), but there were no differences between contexts A, B and C, for both Experiments 4 and 5.

We conclude that [1] it is difficult to draw any conclusions about cerebral (or, even more specifically, hippocampal) glucose levels from peripheral measurements and [2] that, to evaluate the effect of 250 mg/kg glucose administration, it is not advisable to use an interval of 5, 15 or 30 minutes post-administration for peripheral blood glucose measurements with tail vein puncture.

Experiment 6: Materials and Methods

Behavioral procedures were similar to Experiments 1 and 2, except that animals received sham infusions prior to the training session, and muscimol or saline infusions into the dorsal hippocampus (DH) through pre-implanted cannulas prior to the test session, to examine the effect of hippocampal inactivation on the retrieval of generalized contextual fear. We only tested in context A or B, to reduce the number of animals undergoing surgery.

Surgery. Seven or 9 days before training, rats (n = 32) were implanted bilaterally with stainless steel guide cannulas (23G, C317G/ 5 mm, PlasticsOne, USA) aimed at the DH. Rats were anesthetized with ketamine and medetomidine hydrochloride and mounted into the stereotactic frame, where they received additional local anesthesia (lidocaine) before incision. Burr holes were drilled into the skull for the placement of four stainless steel screws and two guide cannulas. The cannulas were placed bilaterally, aimed at the DH (3.8 mm posterior and 3.0 mm lateral to bregma, 1.7 mm subdural), with a 10° angle to the sagittal plane. Dental cement (Tetric EvoFlow, Ivoclar Vivadent, USA) was used to attach the cannulas to the skull and screws. Afterwards, the wound was sutured and the rats were given postoperative pain medication (Metacam, Boehringer Ingelheim Vetmedica, Germany). Dummy cannulas (C317DC/ 5 mm, PlasticsOne) were inserted into the guides to prevent occlusion. Two rats died 4-7 days after surgery.

Infusions. To familiarize the animals with the infusion procedure, rats received sham infusions about 45 min before training. Dummy cannulas were removed and immediately replaced and the animal was attached to the balancing arm of the infusion setup with a collar (CMA 120 System for Freely Moving Animals, CMA Microdialysis, Sweden). Subsequently, the animal was placed inside the infusion cage and the pump (CMA 400 Syringe Pump, CMA Microdialysis) was turned on for 3 minutes. This procedure was repeated for the other hemisphere (in random order, which was reused one day later).
The next day, rats received infusions of muscimol (powder dissolved in saline (1 µg/µl), Sigma-Aldrich, USA) or saline, 44 min (range: 33-48 min) before test. Procedures and dosing are in line with prior research using muscimol infusions to inactivate the dorsal hippocampus[13,14]. Both solutions were prepared in one batch each, kept at -20°C and thawed by hand to room temperature at the start of the test day. Injection cannulas (30G, C317I/ 6 mm, PlasticsOne), which extended 1 mm below the guide cannulas, were inserted into the DH, and muscimol or saline was delivered at an infusion rate of 0.25 µl/min for 2 minutes (0.5 µl per hemisphere). Injection cannulas were left in place for 1 minute after infusion to allow for diffusion. In one rat, which was excluded from analyses, it was impossible to infuse muscimol, because of heavy resistance and some bleeding around the headstage.

Perfusion and histology. Eight days after testing, rats were euthanized and perfused with a sucrose solution and 4% formaldehyde. Brains were fixated and embedded in paraffin. Coronal slices (5 µm) were made and afterwards stained with 0.5% cresyl violet. Cannula positions were verified under a light microscope by two observers, unaware of which rat the sample belonged to. Three rats were excluded because of cannula misplacement, leaving 7 rats in each saline group and 6 rats in each muscimol group.

Experiment 6: Results and Discussion

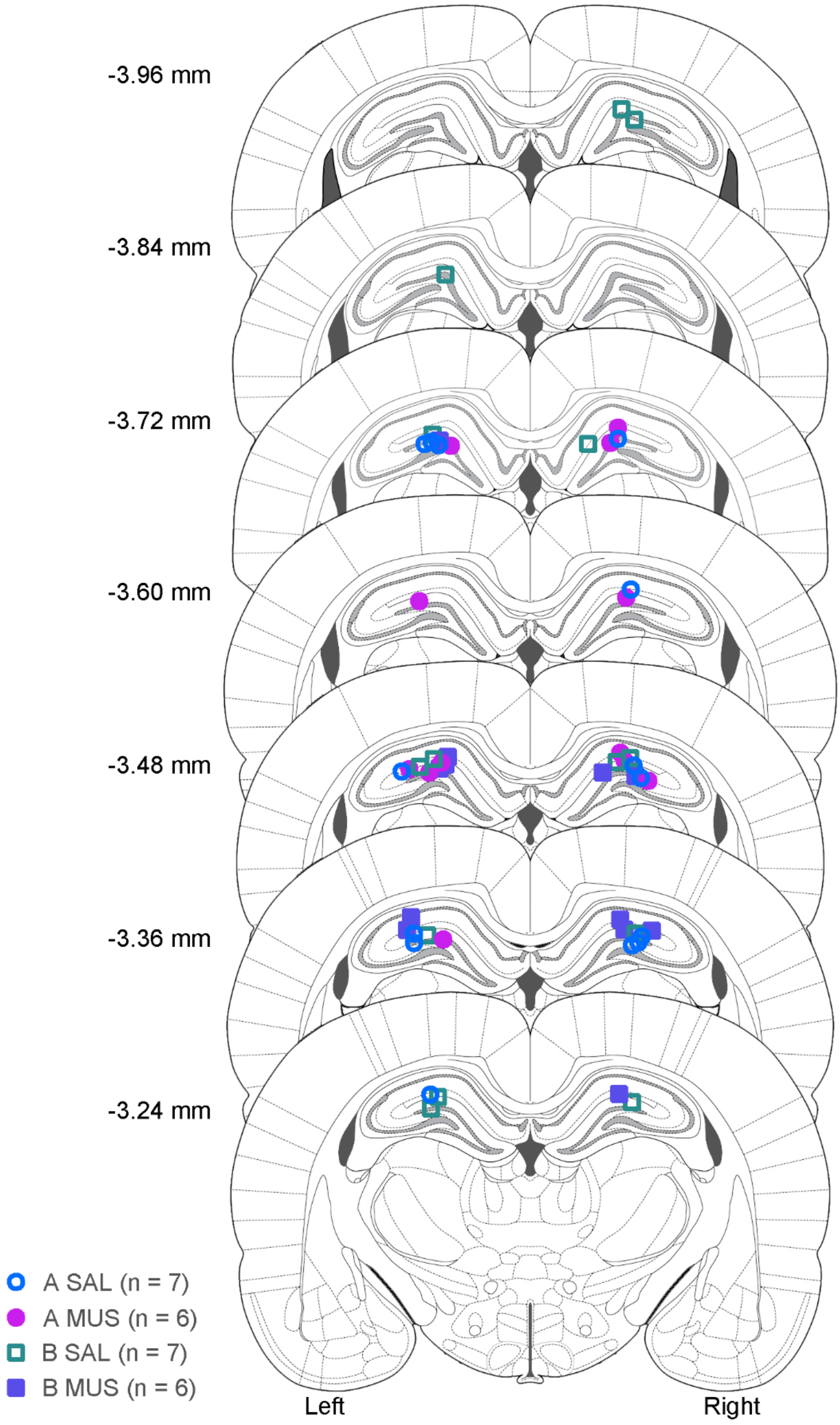

Figure S5: Infusion locations (tip of the injection cannulas) shown on brain diagrams. *Distance posterior to bregma is indicated for each slice. Adapted from[15].*

In this experiment, we aimed to manipulate the dorsal hippocampus directly, as a first step to shed some light on its role in contextual generalization in our protocol. Prior research already implicated the hippocampus in contextual generalization and discrimination[14,16], and recent studies have been scrutinizing this role in more detail. It appears that the hippocampus is necessary for the retrieval of detailed (non-generalized) contextual memories[17], whereas others emphasize its parallel role in generalization[18]. It has been proposed that a combination of young and mature hippocampal neurons contribute to overall memory specificity[19,20]. Furthermore, Glenn and colleagues put forward that their systemic glucose effect might act by ameliorating hippocampal functioning[21].

Fig. S5 shows the location of the tip of the infusion needle in the dorsal hippocampus of

the included rats from all 4 groups. Only rats with bilateral infusions in the dorsal hippocampus were included in the analyses.

A factorial ANOVA showed a main effect of Context ($F(1,22) = 4.87$, $p < .05$), but no main effect of Drug infusion, nor an interaction between both (***Fig. S6***). Tukey's posthoc tests confirmed that the rats discriminated between contexts A and B ($p < .05$), but this was not affected by hippocampal inactivation, although we used standard muscimol procedures[13,14].

Note that freezing in this experiment was much lower than in Experiments 1-5 with unoperated animals. In those studies, post-shock freezing was 45% ± 18% (mean ± SD, n = 181), freezing during test in context A was 65% ± 20% (n = 39, i.e. only including control animals) and in context B 40% ± 20% (n = 39). With operated animals, post-shock freezing dropped to 25% ± 18%, while freezing during test in the control groups was only 22% ± 25% in context A and 7% ± 9% in context B. This might reflect a non-specific effect of surgery on the propensity to freeze. Such low levels were unexpected, as we and others have previously used recovery times of 7-9 days without problems[13,22], but may be overcome by a longer recovery period. Nevertheless, for the current experiment, it does hamper the interpretation of any effects of muscimol.

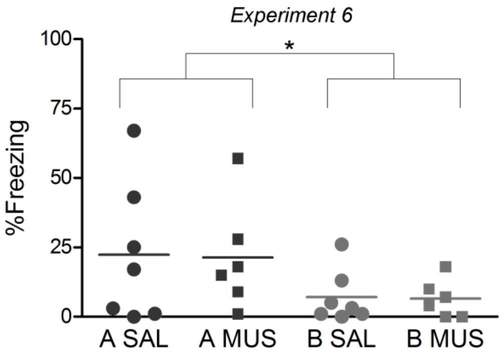

Figure S6: Effect of pre-test hippocampal inactivation on contextual generalization*. %Freezing during the 8-min test in Experiment 6. Because of small group sizes (n = 6-7 per group), individual data are shown. Group means are indicated by horizontal lines. There is no effect of hippocampal inactivation with muscimol, but we do replicate the discrimination between contexts A and B (SAL and MUS rats pooled, n = 13 per context), *p<.05, Tukey's posthoc test. A SAL = saline rats tested in context A; A MUS = muscimol rats tested in context A; B SAL = saline rats tested in context B and B MUS = muscimol rats tested in context B.*

We did not find evidence for a reduction of contextual freezing in context A after taking the hippocampus offline[23-27]. Although unexpected, this is consistent with other findings[14].
Because of the low overall freezing levels, it was difficult to evaluate the effect of pre-test hippocampal inactivation in context B in our generalization procedure. Prior research found that pretest dorsal hippocampus inactivation reduced freezing in context A, 15 days after training, but only in mice that were classified as 'discriminators', not in 'generalizers'[17]. The authors did not test hippocampal inactivation in generalization context B. An earlier study investigated the effect of complete hippocampal lesions (NMDA) made 1 day after discrimination training in shock context A and no-shock context B[28]. Eight days after training, freezing of mice with lesions was reduced in context A, but unaffected in context B. Very recently, a mouse experiment with a 21-day interval (a 1-day interval was not tested) between training and test found that pretest dorsal hippocampus inactivation (lidocaine) reduced freezing in context A, but had no effect on freezing in context B[29]. Taken together, it was difficult to predict which effect hippocampal inactivation would have in our contextual generalization protocol, as prior research underscores that the effects of hippocampal manipulations are highly dependent on the timing of the manipulation with regard to training and test, the interval between these two, the applied lesion or inactivation technique, the precision or quality of the contextual memory and even individual differences between animals.

Supplementary References

S1. Luyten L, Fanselow MS, Vansteenwegen D, Nuttin B, Hermans D. Broad contextual generalization gradients in rats - Optimization of a behavioral protocol. Scientific Advisory Board Meeting of the Center for Excellence on Generalization Research, Leuven, Belgium 2013.

S2. Luyten L, Casteels C, Vansteenwegen D, van Kuyck K, Koole M, Van Laere K, Nuttin B. Micro-positron emission tomography imaging of rat brain metabolism during expression of contextual conditioning. J Neurosci 2012;32:254-63.

S3. Luyten L, Vansteenwegen D, van Kuyck K, Deckers D, Nuttin B. Optimization of a contextual conditioning protocol for rats using combined measurements of startle amplitude and freezing: the effects of shock intensity and different types of conditioning. J Neurosci Methods 2011;194:305-11.

S4. McIntyre N, Holdsworth CD, Turner DS. New Interpretation of Oral Glucose Tolerance. Lancet 1964;2:20-1.

S5. Teff KL. How neural mediation of anticipatory and compensatory insulin release helps us tolerate food. Physiol Behav 2011;103:44-50.

S6. Hui IR, Hui GK, Roozendaal B, McGaugh JL, Weinberger NM. Posttraining handling facilitates memory for auditory-cue fear conditioning in rats. Neurobiol Learn Mem 2006;86:160-3.

S7. Schwabe L, Schachinger H, de Kloet ER, Oitzl MS. Corticosteroids operate as a switch between memory systems. J Cogn Neurosci 2010;22:1362-72.

S8. Marquez C, Nadal R, Armario A. The hypothalamic-pituitary-adrenal and glucose responses to daily repeated immobilisation stress in rats: individual differences. Neuroscience 2004;123:601-12.

S9. Wong KP, Sha W, Zhang X, Huang SC. Effects of administration route, dietary condition, and blood glucose level on kinetics and uptake of 18F-FDG in mice. J Nucl Med 2011;52:800-7.

S10. Ghezzi AC, Cambri LT, Botezelli JD, Ribeiro C, Dalia RA, de Mello MA. Metabolic syndrome markers in wistar rats of different ages. Diabetol Metab Syndr 2012;4:16.

S11. de Vries MG, Arseneau LM, Lawson ME, Beverly JL. Extracellular glucose in rat ventromedial hypothalamus during acute and recurrent hypoglycemia. Diabetes 2003;52:2767-73.

S12. Dunn-Meynell AA, Sanders NM, Compton D, Becker TC, Eiki J, Zhang BB, Levin BE. Relationship among brain and blood glucose levels and spontaneous and glucoprivic feeding. J Neurosci 2009;29:7015-22.

S13. Corcoran KA, Desmond TJ, Frey KA, Maren S. Hippocampal inactivation disrupts the acquisition and contextual encoding of fear extinction. J Neurosci 2005;25:8978-87.

S14. Holt W, Maren S. Muscimol inactivation of the dorsal hippocampus impairs contextual retrieval of fear memory. J Neurosci 1999;19:9054-62.

S15. Paxinos G, Watson C. The rat brain in stereotaxic coordinates. 6th ed. Amsterdam: Academic Press/Elsevier; 2007.

S16. Gerlai R. Contextual learning and cue association in fear conditioning in mice: a strain comparison and a lesion study. Behav Brain Res 1998;95:191-203.

S17. Wiltgen BJ, Zhou M, Cai Y, Balaji J, Karlsson MG, Parivash SN, Li W, Silva AJ. The hippocampus plays a selective role in the retrieval of detailed contextual memories. Curr Biol 2010;20:1336-44.

S18. Kumaran D. What representations and computations underpin the contribution of the hippocampus to generalization and inference? Front Hum Neurosci 2012;6:157.

S19. Aimone JB, Deng W, Gage FH. Resolving new memories: a critical look at the dentate gyrus, adult neurogenesis, and pattern separation. Neuron 2011;70:589-96.

S20. Nakashiba T, Cushman JD, Pelkey KA, Renaudineau S, Buhl DL, McHugh TJ, Rodriguez Barrera V, Chittajallu R, Iwamoto KS, McBain CJ, Fanselow MS, Tonegawa S. Young dentate granule cells mediate pattern separation, whereas old granule cells facilitate pattern completion. Cell 2012;149:188-201.

S21. Glenn DE, Minor TR, Vervliet B, Craske MG. The effect of glucose on hippocampal-dependent contextual fear conditioning. Biol Psychiatry 2014;75:847-54.

S22. Luyten L, van Kuyck K, Vansteenwegen D, Nuttin B. Electrolytic lesions of the bed nucleus of the stria terminalis disrupt freezing and startle potentiation in a conditioned context. Behav Brain Res 2011;222:357-62.

S23. Fanselow MS. Contextual fear, gestalt memories, and the hippocampus. Behav Brain Res 2000;110:73-81.

S24. Kim JJ, Fanselow MS. Modality-specific retrograde amnesia of fear. Science 1992;256:675-7.

S25. de Oliveira Alvares L, Einarsson EÖ, Santana F, Crestani AP, Haubrich J, Cassini LF, Nader K, Quillfeldt JA. Periodically reactivated context memory retains its precision and dependence on the hippocampus. Hippocampus 2012;22:1092-5.

S26. McNish KA, Gewirtz JC, Davis M. Evidence of contextual fear after lesions of the hippocampus: a disruption of freezing but not fear-potentiated startle. J Neurosci 1997;17:9353-60.

S27. Frankland PW, Cestari V, Filipkowski RK, McDonald RJ, Silva AJ. The dorsal hippocampus is essential for context discrimination but not for contextual conditioning. Behav Neurosci 1998;112:863-74.

S28. Wang SH, Teixeira CM, Wheeler AL, Frankland PW. The precision of remote context memories does not require the hippocampus. Nat Neurosci 2009;12:253-5.

S29. Cullen PK, Gilman TL, Winiecki P, Riccio DC, Jasnow AM. Activity of the anterior cingulate cortex and ventral hippocampus underlie increases in contextual fear generalization. Neurobiol Learn Mem 2015.
